# Mobile- and Web-Based Interventions for Promoting Healthy Diets, Preventing Obesity, and Improving Health Behaviors in Children and Adolescents: Systematic Review of Randomized Controlled Trials

**DOI:** 10.2196/60602

**Published:** 2025-05-20

**Authors:** Clara Talens, Noelia da Quinta, Folasade A Adebayo, Maijaliisa Erkkola, Maria Heikkilä, Kamilla Bargiel-Matusiewicz, Natalia Ziółkowska, Patricia Rioja, Agnieszka E Łyś, Elena Santa Cruz, Jelena Meinilä

**Affiliations:** 1 AZTI Food Research, Basque Research and Technology Alliance (BRTA) Derio Spain; 2 Department of Food and Nutrition Faculty of Agriculture and Forestry University of Helsinki Helsinki Finland; 3 Faculty of Psychology University of Warsaw Warsaw Poland; 4 Faculty of Psychology and Cognitive Science Adam Mickiewicz University in Poznań Poznań Poland

**Keywords:** nutrition education, digital health, serious games, mHealth, eHealth, childhood obesity prevention, physical activity promotion, dietary behavior, mobile apps, web-based intervention, nutrition literacy, school health, gamified intervention, adolescent health, randomized controlled trial

## Abstract

**Background:**

Childhood and adolescent obesity is a growing global health issue linked to noncommunicable diseases such as cardiovascular disease and type 2 diabetes. Digital health technologies, including mobile apps and web-based programs, offer scalable tools to improve health behaviors, but their effectiveness in young populations remains unclear.

**Objective:**

This systematic review aimed to evaluate the effectiveness of mobile and web-based digital interventions in promoting healthy diets, reducing obesity risk, increasing physical activity, and improving nutrition-related knowledge and attitudes among children and adolescents.

**Methods:**

A systematic search was conducted across PubMed, Scopus, Web of Science, and Google Scholar databases, along with hand-searching reference lists of key systematic reviews. The search encompassed records published up to September 30, 2024. Eligible studies were randomized controlled trials targeting dietary intake, anthropometric measurements, physical activity, or nutrition-related attitudes and knowledge among participants aged ≤18 years. Screening, full-text eligibility assessment, and data extraction were done partly in duplicate (20%; κ=0.86 for title or abstract screening, κ=0.71 for full-text eligibility assessment, and κ=0.78 data extraction). Risk of bias was evaluated using the Cochrane Risk-of-Bias tool (κ=0.71 for interrater reliability of 20% duplicate evaluation). Data were synthesized narratively.

**Results:**

From 300 records screened, a total of 37 articles (34 studies) were included. Interventions included games, (in 21/34 studies, 62%), mobile apps, web-based programs, and other digital tools. Among the 34 included studies, 23 (68%) studies reported positive outcomes for at least 1 measured variable. Fruit intake improved in 17 of 34 studies (50%) assessing fruit intake, while 7 of 34 studies (21%) targeting sugar-sweetened beverage consumption showed reductions. Improvements in nutrition knowledge were reported in 23 of 34 (68%) studies, but changes in anthropometric measures and physical activity outcomes showed no effect. Risk of bias was low for random sequence generation but high or unclear in other domains for many studies.

**Conclusions:**

Mobile- and web-based interventions, particularly game-based tools, show promise for promoting healthy dietary behaviors and increasing nutrition knowledge in children and adolescents. However, the evidence for long-term sustainability and impact on anthropometric and physical activity outcomes remains limited. Future research should focus on understanding which digital features drive effectiveness, extending follow-up periods, and exploring the role of family involvement in interventions.

**Trial Registration:**

PROSPERO CRD42023423512; https://www.crd.york.ac.uk/prospero/display_record.php?RecordID=423512

## Introduction

### Background

The World Health Organization recognizes overweight and obesity as a global epidemic affecting both low- and middle-income countries and higher-income countries [1,2]. They pose a substantial threat and challenge to public health, as they are also linked to the efficiency of the health care system [1,3]. In particular, obesity and overweight are associated with health effects and diseases in childhood and adolescence. The literature indicates that such risks include metabolic syndrome, type 2 diabetes, and hypertension [1,4]. Global research data are alarming, as they show that as many as 39 million children younger than 5 years were living with overweight or obesity in 2020, and more than 340 million children and adolescents aged 5 to 19 years were diagnosed with overweight or obesity in 2016 [5].

In addition, children consume fruits and vegetables at levels well below the recommended values. Furthermore, more than 80% of adolescents worldwide do not engage in sufficient physical activity [6,7]. A lack of a well-balanced diet combined with little or no physical activity may contribute to the development of noncommunicable diseases such as cancer, diabetes, and cardiovascular disease [8,9].

Childhood obesity can also have severe psychological effects, as studies have shown that childhood obesity affects self-esteem and is associated with depression, lower quality-of-life outcomes, and emotional and behavioral difficulties [10]. In addition, diminished life satisfaction—an important dimension of subjective well-being—is associated with overweight and obesity [11] and low subjective health perceptions [12]. Obesity also contributes to poor functioning in the peer environment in children, as children with obesity report stigmatization and bullying by peers [10].

It is necessary to develop a coherent strategy to combat the obesity epidemic among children and adolescents, as obesity poses a threat to the health and well-being of individuals and has an impact on resources and economic costs [10]. Health education can foster and reinforce healthy eating habits and attitudes through various interventions aimed at children and adolescents.

In children and adolescents, specially developed games and apps may help develop and promote healthy lifestyles [8]. Gamification uses the process of teaching or learning with new technologies and apps, which are very attractive to this age group. The games are designed to promote motivation for various achievements and awards but also include elements of interaction between the participants [13,14]. Elements of gamification have been used to improve the uptake of healthy habits (such as increased physical activity or improved nutrition) [15,16], to reinforce changes in habits and behaviors [17,18], and to enhance positive emotions associated with subjective well-being [17,19]. There is some evidence that after game-based interventions among children and adolescents, fruit and vegetable consumption increase, but the improvement in nutrition-related knowledge [20-23] is inconsistent.

### Objectives

The aim of this paper was to present conclusions from a systematic review (SR) of scientific literature on the effectiveness of mobile- and web-based interventions in promoting healthy diets, preventing obesity, promoting physical activity, and improving attitudes and knowledge toward nutrition in children and adolescents compared with traditional interventions and a lack of any intervention. The findings might constitute the basis for planning methodologically sound subsequent mobile- and web-based interventions for promoting health in children and adolescents.

Unlike previous SRs that focused on either dietary or physical activity outcomes, this review provided a comprehensive synthesis of digital interventions targeting multiple health behaviors in children and adolescents. Given the increasing integration of digital tools that address multiple lifestyle behaviors simultaneously, our broad inclusion criteria allowed us to capture real-world applications of these interventions. This approach provided valuable insights for designing future interventions that consider the combined impact of diet and physical activity on health outcomes.

## Methods

### Protocol and Registration

The review was registered with PROSPERO, with the registration number CRD42023423512. PRISMA (Preferred Reporting Items for Systematic Reviews and Meta-Analyses) 2020 statement was used for reporting this review [24]. There were no deviations from the registered protocol. A meta-analysis was not planned from the outset due to the anticipated heterogeneity of interventions.

### Information Sources

A systematic literature search was conducted across 4 databases, including Web of Science, PubMed, Scopus, and Google Scholar, to identify relevant randomized controlled trials (RCTs) on mobile- and web-based interventions for promoting healthy diets, preventing obesity, and improving health behaviors in children and adolescents. The search encompassed records available up to September 30, 2024.

### Search Strategy

The search strategy was developed collaboratively by the research team, with the guidance of an experienced librarian. The librarian provided expertise in constructing comprehensive and precise search strings. Keywords were identified based on an iterative review of the existing literature, discussions among team members, and previous SRs in related fields. The selection of terms reflected the study objectives, focusing on key elements such as population (eg, children and adolescents), interventions (eg, games and digital tools), and outcomes (eg, dietary habits and physical activity). Search terms were tested and refined to ensure they captured a broad yet relevant evidence base, with final search strings tailored for each database (PubMed, Web of Science, Scopus, and Google Scholar). The search words focused on the target population, intervention types, and desired outcomes, using Boolean operators for precise search queries. The search queries per database are presented in Multimedia Appendix 1, and the results from each database query are provided in Multimedia Appendix 2.

SRs identified during the database search were excluded from the primary analysis as they did not meet the inclusion criteria related to study type. However, to ensure comprehensiveness, all SRs identified were reviewed for their relevance to include original studies that aligned with our scope. Reference lists of SRs that were relevant in terms of our research questions were reviewed for relevant original articles. Duplicate records were identified and removed using the web-based SR software Covidence (Veritas Health Innovation Ltd). The process involved automatic detection of duplicates followed by manual verification to ensure accuracy. Any discrepancies in duplicate identification were resolved through discussion among the reviewers.

### Eligibility Criteria

Articles were included when the outcomes of interest concerned dietary intake, diet quality, anthropometric measurements (such as BMI or weight), physical activity, or attitudes and knowledge related to diet or food. We included studies with a mean age of the participants from 6 months to 18 years and with participants without a preexisting severe chronic disease. Nonrandomized studies and case reports were excluded. Articles in languages other than English, Spanish, Finnish, or Polish were excluded because of limited resources for translation. However, no studies in other languages appeared in the search.

### Screening

The first screening was carried out using the population, intervention, comparison, outcomes, study design, and time (PICOST) framework [25]. To ensure a systematic and comprehensive approach, the PICOST framework was used to inform both the development of the search query and the inclusion or exclusion criteria. The population focused on children and adolescents (eg, *children*, *adolescents*, *kids*, and *family*), targeting studies that investigated participants with a mean age ≤18 years. The intervention criteria included digital and mobile-based tools promoting health, such as game-based interventions (*games for health*, *mobile phone*, *digital tools*, and *gamification*). The comparison element was not explicitly restricted, allowing studies comparing digital interventions with traditional methods, no intervention, or other digital strategies. The outcomes criteria were centered on dietary behaviors, nutrition, and health-related habits, as reflected in the search terms *dietary habits*, *food habits*, *dietary choices*, *healthy eating*, *eating behavior*, and *healthy lifestyle*. These outcomes were aligned with public health objectives aiming to track improvements in eating behaviors and nutritional intake. The study design criteria focused on RCTs evaluating the effectiveness of these interventions. Finally, while the time criteria were flexible, preference was given to studies that reported both short-term and long-term outcomes. This structured approach guided our search, leading to the use of Boolean operators and specific keywords to capture relevant studies in the databases, as reflected in the search queries. A detailed list of inclusion and exclusion criteria used during the screening process is provided in Multimedia Appendix 3.

The titles and abstracts of the studies were independently screened by 5 authors (CT, NdQ, FAA, NZ, and PR), with each author reviewing a subset of the results. To assess interrater reliability, 20% (65/324) of the studies were screened in duplicate by 2 independent reviewers, and the κ coefficient was calculated to evaluate the consistency of the screening process. The agreement between the duplicate selections was found to be high (κ=0.86), and therefore, the rest of the selections were performed by 1 author. The conflicts in the 20% duplicate selection were resolved through discussion by the researchers concerned.

Following the title and abstract screening, full-text articles of studies that met the initial inclusion criteria were retrieved and assessed for eligibility. The full-text eligibility assessment was conducted by single reviewers for most articles, with 20% (18/89 full texts) assessed in duplicate to ensure consistency (κ=0.71 for interrater agreement). Discrepancies in duplicate assessments were resolved through discussion among reviewers.

### Data Extraction

The data were extracted using the web-based SR software Covidence. To extract the data from the primary articles, we used a data extraction form (Multimedia Appendix 4) adapted from the one used by the Cochrane Collaboration [26]. Five authors (ESC, FAA, NZ, CT, and NdQ) extracted the data. After the first round of data extraction for approximately 19% (7/37) of the records, the data were independently reviewed by another author (ESC, FAA, NZ, CT, and NdQ). At this level of screening, the interrater reliability was *κ*=0.71. Disagreements were resolved through discussion, and if consensus could not be reached, a third reviewer was consulted.

The extracted data encompassed a comprehensive set of information, including but not limited to general characteristics (authors, publication year, and study country), study population or sample details, aims, intervention characteristics, study duration, and various types of outcome measures. The results were synthesized narratively because the interventions had too many differences compared with each other. The results were grouped for reporting according to the outcome. All types of effect measures (risk ratio, odds ratio, means, among others) were extracted.

### Effectiveness of Interventions

The effectiveness of the interventions was evaluated based on the difference between the intervention and control groups as a result of the intervention in the outcomes of interest (Eligibility Criteria section).

### Risk-of-Bias Assessment

The risk of bias was assessed using the Cochrane Collaboration Risk-of-Bias assessment tool [26], which evaluates 6 domains: random sequence generation, allocation concealment, blinding of participants and personnel, blinding of outcome assessment, incomplete outcome data, and selective reporting. Each domain was rated as low, high, or unclear risk. The assessment was conducted in duplicate for 20% of the included studies to ensure reliability (κ=0.71). Disagreements were resolved through discussion and, if necessary, consultation with a third reviewer.

## Results

### Included Studies

**Figure 1:**
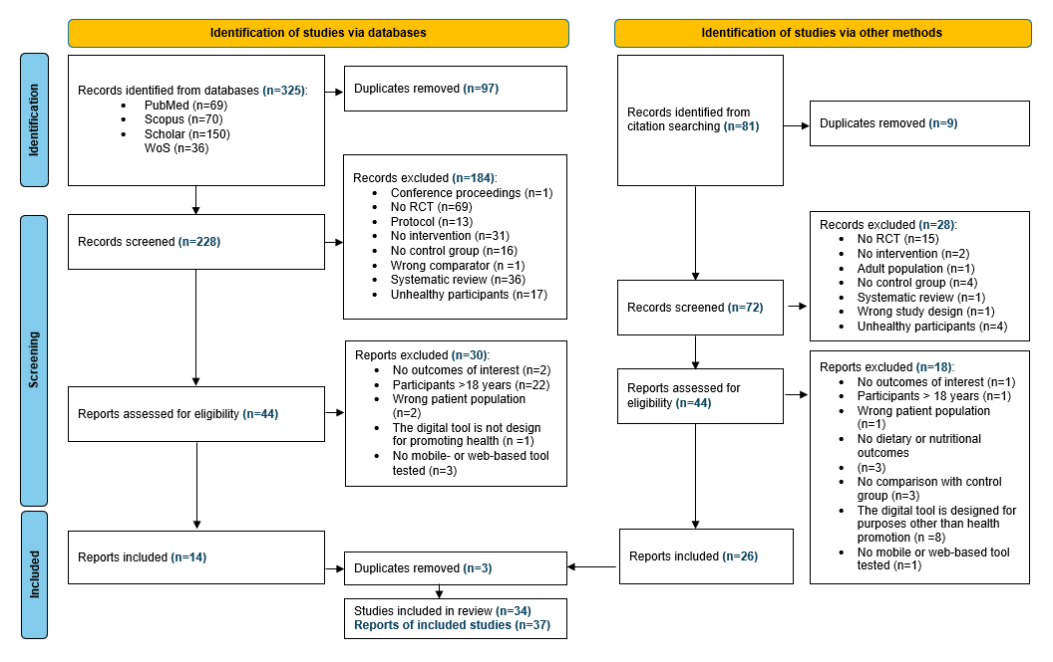
Study selection following the PRISMA (Preferred Reporting Items for Systematic Reviews and Meta-Analyses) criteria detailing the study selection process. RCT: randomized controlled trial.

The database search resulted in 325 records, and 228 after removing duplicates (Figure 1).

Of the 36 SRs identified, 4 were considered relevant to our research questions, the rest were excluded. From the reference list of the 4 relevant SRs, 81 articles were identified as relevant.

After removing duplicates, 72 were screened [5,8,27,28]. Altogether 300 articles were screened from which 88 were rigorously assessed for eligibility. In total, 37 articles based on 34 intervention studies were found to be eligible. Of the eligible RCTs, 3 reported their results in 2 publications [29-34]. The number of excluded studies and reasons for exclusion are listed in Figure 1.

### Characteristics of the Interventions

The included studies, published between 2003 and 2024, evaluated digital interventions targeting dietary behaviors, physical activity, and other lifestyle-related behaviors in children and adolescents. The interventions varied in design, duration, and behavioral focus, with some addressing multiple lifestyle behaviors simultaneously The characteristics of the included studies and a detailed description of the interventions are summarized in Table 1 and Table 2, respectively.

**Table 1:** Characteristics of the included studies.

Study, year, country	Sample	Aim	Setting and intervention	Duration of treatment period	Main and secondary outcomes	Types of outcome measures
Bannon and Schwartz [35], 2006, United States	N=50;age 5 y; CG^a^: n=18; IG^b^: n=14 (gain framed), n=18 (loss framed)	To investigate the influence of nutrition message framing on snack choice among kindergartners, specifically examining the effectiveness of gain-framed and loss-framed messages in promoting healthy snack choices among children. The study sought to determine the impact of message framing on children’s behavior and their perceptions of healthy versus unhealthy foods.	School CG: no videos. Control condition as a comparison. IG: compared the effects of gain-framed and loss-framed videos on children’s food choices using a control condition as a comparison.	3 d	Healthy food choices	Dietary intake
Baños et al [36], 2013, Spain	N=228; age 10-13 y; CG: n=155; IG: n=73	To assess the efficacy and acceptability of a web-based game platform called ETIOBE Mates in improving children’s nutrition knowledge	School CG: paper–pencil intervention IG: ETIOBE Mates intervention	2 wk	Knowledge on nutrition	Knowledge
Baranowski et al [37], 2003, United States; Cullen et al [38], 2005, United States	N=1578; age 8-12 y; CG: n=793; IG: n=785	To demonstrate dietary change immediately after implementation of the Squire’s Quest! program	School CG: received no food education intervention during the study period IG: received Squire’s Quest!	5 wk, each session lasting 25 min	Dietary intake of vegetables, high-fat vegetables, fruit, juice, fruit, juice, and vegetables (PO^c^) fruit, juice, and vegetables with high-fat vegetables	Dietary intake (PO)
Baranowski et al [39], 2011, United States	N=133;age 10-12 y; CG: n=40; IG: n=93	To evaluate outcome from playing Escape from Diab (Diab) and Nanoswarm: Invasion from Inner Space (Nano) video games on children’s diet, physical activity, and adiposity.	School CG: provided with an internet experience that included a booklet and 2 discs. The first disc contained links to 8 sessions of game-based websites with health-related content, along with questions to answer postsession for immediate feedback. The second disc included a knowledge-based nutrition game (Good Food and Play Make a Balanced Day for Diab and Dish It Up for Nano) played alongside the 8 session websites. IG: played Escape from Diab and Nanoswarm: Invasion from Inner Space video games. Each game included 9 sessions with sequential educational content and knowledge minigames focused on behavior change.	9 sessions, with a minimum of 40 min of gameplay per session, 6 h of new gameplay per game	Dietary intake of fruit, juice and vegetables, water, total energy intake, sedentary time per day, frequency of light activity, frequency of moderate-to-vigorous activity	Dietary intake and physical activity
Byrne et al [40], 2012, United States	N=39;age 12-14 y; CG: n=13; IG: n=13 (positive-negative), n=13 (positive only)	To test efficacy of mobile technologies to motivate adolescents to make healthy nutritional choices by making them interact with a virtual pet game that responds to their breakfast behaviors	School CG: no pet IG: (1) the positive and negative feedback condition (positive-negative) and (2) the positive or neutral only feedback condition (positive only) Virtual pet versus no pet conditions	9 d	Frequency of having breakfast, healthy eating	Dietary intake, attitude or intrinsic motivation
Carlin et al [41], 2021, United Kingdom (Northern Ireland)	Phase 1: N=11 families; age 5-12 y; CG: n=5; IG: n=6. Phase 2: N=15 families; age 5-12 y; CG: n=7; IG: n=8	To promote positive health behaviors in the family setting through the use of the functions of a smart speaker and its linked intelligent personal assistant	Home CG: continue as usual without the provision of additional technology IG: intelligent personal assistant	12 wk	Active time per day, sedentary time per day	Physical activity
Chagas et al [42], 2020, Brazil	N=319; age 13-19 y; CG: n=202; IG: n=117	To assess the individual-level impact of a nutritional intervention for high school students on food consumption, nutrition knowledge, and self-efficacy in the adoption of healthy eating practices based on the use of a digital game that was developed to promote healthy eating	School CG: neither game nor recommendations IG: played Rango Cards game	From 7 (minimum) to 17 (maximum) d	Knowledge on nutrition and healthy eating	Knowledge
Clarke et al [43], 2019, United States	N=149 participants per 15 pantries; age 9-14 y; CG: n=47 participants per 6 pantries; IG: n=102 participants per 9 pantries	To test the effectiveness of 1app by determining whether it increased the use of vegetables in preparations of meals and snacks compared with a control group	Food pantry CG: no mobile app IG: smartphone with app and a phone use plan, then trained to use the app	5 wk	Dietary intake of target vegetable preparations (PO), general vegetable preparations	Dietary intake (PO)
de Vlieger et al [44], 2021, Australia	N=169; age 9-12 y; CG: n=94; IG: n=75	To investigate the feasibility and acceptability of VitaVillage as a nutrition education tool in primary schools. In addition, the effectiveness of the game in increasing children’s short-term nutrition knowledge was explored	School CG: no nutrition education IG: played VitaVillage game	Baseline T1 and after 1 wk T2	Knowledge on nutrition	Knowledge
Espinosa-Curiel et al [22], 2020, Mexico	N=27;age 9.9 y; CG: n=12; IG: n=15	To promote healthy lifestyle behaviors (physical activity, healthy eating, and socioemotional wellness) in children using the serious game HelperFriend	School CG: 45-min talk about the importance of healthy behaviors, such as engaging in physical activity, eating healthily, and maintaining socioemotional health IG: played the HelperFriend game during six 30-min sessions	4 wk	Knowledge on healthy eating	Knowledge
Fassnacht et al [45], 2015, Germany	N=49;age 9.6 y; CG: n=27; IG: n=22	To explore the efficacy of using mobile phone SMS text messaging to promote health behaviors in school-aged children	School CG: no monitoring. Control condition as a comparison IG: participants reported their health behaviors (fruit and vegetable consumption, physical activity, screen time) daily using SMS text messaging and received supportive feedback for more than 8 wk	8 wk	Dietary intake of fruit and vegetables, active time per day	Dietary intake and physical activity
Folkvord et al [46], 2013, Netherlands	N=270; age 8-10 y; CG: n=69; n (IG1)=69; n (IG2)=67; n (IG3)=65; n (IGT)=201	To examine the effect of advergames that promote energy-dense snacks or fruit on children’s ad libitum snack and fruit consumption and whether this consumption differed according to brand and product type (energy-dense snacks and fruit). The second aim was to examine whether advergames can stimulate fruit intake.	School CG: did not play a game IG1: played an advergame that promoted energy-dense snacks IG2: played an advergame that promoted fruit IG3: played an advergame that promoted nonfood products	5 min	Dietary intake of fruit, snacks or energy-dense snacks, jelly candy, banana, apple, chocolate, total food intake in 1 meal; physical activity and active time per day	Dietary intake and physical activity
Folkvord et al [47], 2021, Netherlands	N=157; age 6-12 y; n (CG)=62; n (IG)=95	To test the effectiveness of a serious health game that was specifically developed to improve eating behavior among children	School CG: children attended regular classes and did not play a game IG: children played a serious game	1 wk	Dietary intake of jelly candy, banana, mandarin, and chocolate. Attitude toward banana, mandarin, jelly candy, and chocolate	Dietary intake and attitude or intrinsic motivation
Froome et al [48], 2020, Canada	N=95; age 8-10 y; n (CG)=34; n (IG)=39	To determine the efficacy of the Foodbot Factory mobile app in improving children’s knowledge of Canada’s food guide. The study specifically compared the effectiveness of the Foodbot Factory app against a control group using another app. It focused on assessing significant improvements in overall nutrition knowledge and specific subscores related to various food groups.	School and home CG: used My Salad Shop Bar, a cooking game focusing on preparing healthy foods IG: used Foodbot Factory, a serious game designed to teach about Canada’s food guide	5 d	Knowledge on nutrition (PO), vegetables and fruit, protein foods, whole-grain foods, and drinks	Knowledge (PO)
Gan et al [49], 2019, Philippines	N=360; age 7-10 y; n (CG)=180; n (IG)=180	To determine the effectiveness of Healthy Foodie on the nutrition knowledge of children aged 7-10 y	School CG: children did not play Healthy Foodie game IG: children played Healthy Foodie game	30 min	Knowledge on food groups and food frequencies and knowledge on nutrition	Knowledge
Haddad et al [50], 2023, Switzerland	N=24;age 8 y; n (CG)=12; n (IG)=12	To assess the feasibility of a home-based cooking intervention using a smartphone app to improve dietary behavior and food acceptability in children aged 7-9 y	Home CG: children did not participate in cooking the target recipes (wholemeal pasta and brussels sprouts) IG: children were involved in cooking the target recipes with limited parental support more than a 10-wk period	10 wk	Dietary intake of brussels sprout and wholemeal pasta; attittude toward food liking	Dietary intake and attitude or intrinsic motivation
Hammersley et al [51], 2019, Australia	N=86; age 2-5 y; n (CG)=44; n (IG)=42	To assess the efficacy of a parent-focused, internet-based healthy lifestyle program for preschool-aged children on child BMI, obesity-related behaviors, parent modeling, and parent self-efficacy	Home, community setting CG: participants received email communication only IG: participants received an 11-wk internet-based healthy lifestyle program followed by fortnightly emails for 3 mo. They also accessed a closed Facebook group to communicate with other participants and a dietitian	6 mo	BMI (PO) dietary intake of vegetables, fruit, discretionary foods, sugar or sucrose, saturated fat. Physical activity (active time per day, sedentary time per day, frequency of light activity, frequency of moderate physical activity, frequency of vigorous physical activity. Attitude toward child feeding practices	Anthropometric measurements (PO), dietary intake, physical activity, attitude or intrinsic motivation
Heikkilä et al [52], 2019, Finland	N=79; age 16-20 y; n (CG)=37; n (IG)=42	To investigate whether young Finnish endurance athletes’ nutrition knowledge and dietary intake can be improved through an education intervention with or without a mobile food app. The primary aim of this study was to create a new scalable, flexible education program to improve nutrition knowledge among young Finnish endurance athletes.	Home CG: participatory nutrition education sessions alone IG: use of a mobile food app for 4 d after each session	17 wk	Dietary intake of carbohydrates, fat, protein, total energy intake. Knowledge on nutrition (PO), nutrition recommendations for endurance athletes, dietary supplements, fluid balance and hydration, energy intake and recovery, association between food choices and body image	Dietary intake and knowledge (PO)
Hermans et al [53], 2018, Netherlands	N=108;age 10-13 y; n (CG)=50; n (IG)=58	To test the short-term effectiveness of the Alien Health Game, a video game designed to teach elementary schoolchildren about nutrition and healthy food choices	School CG: played a web-based nutrition game IG: played Alien Health Game using the Kinect sensor	2 wk	Dietary intake of nutrient-dense food, energy-dense food, sugar, or sucrose. Knowledge on macronutrients. Macronutrient function, Healthier food items	Dietary intake and knowledge (PO)
Kato-Lin et al [54], 2020, India	N=104; age 10-11 y; n (CG)=52; n (IG)=52	To (1) examine the immediate impact of a pediatric dietary mobile game with implicit learning on children’s actual food choices, (2) quantify children’s heterogeneous gameplay patterns, and (3) understand the effects of game engagement by associating gameplay patterns with players’ actual food choices	Home CG: no food education. IG: food education.	1 wk	Dietary intake of healthy food choices	Dietary intake
Mack et al [55], 2020, Germany	N=82; age 9-12 y; n (CG)=42; n (IG)=40	To evaluate the newly developed game (Kids Obesity Prevention) and how well children are able to understand and apply the (DED-P^d^)	School and home CG: received basic information about a healthy lifestyle via a brochure called So macht Essen Spaß (How to Make Food Fun) handed out to the children at the beginning of the study phase IG: played the Kids Obesity Prevention game	2-wk period	Healthy nutrition index, physical activity score, food pyramid score, dietary energy density score	Dietary intake, physical activity, and knowledge (only secondary outcomes were relevant for this review)
Marsh et al [56], 2015, New Zealand	N=78; age 13-18 y; n (CG)=39; n (IG)=39	To compare total energy intake between adolescent males and females aged 13-18 y with access to either a single screen or multiple screens, in the absence of television advertising	Study clinic CG: single-screen group (television) IG: multiscreen group (iPad+mobile phone+television)	3 mo	Total energy intake (PO): from drinks, from high-energy density foods, from low-energy density foods, from M&M, from potato chips, from popcorn, from apple and from yogurt	Dietary intake (PO)
Nezami et al [57], 2018, United States	N=51; age 3-5 y; n (CG)=27; n (IG)=24	To assess the effect of the Smart Moms mobile-based intervention on reducing child SSB^e^ and juice intake, with mothers as the agents of change, while providing weight loss content for the mothers	Home CG: participants did not receive any intervention for 6 mo but received a modified version of the program after the 6-mo assessments IG: participants were asked to reduce their child’s SSB or juice intake to no more than 4 oz per d, reduce their own caloric beverage consumption to no more than 8 oz per d, and submit daily self-monitoring texts for more than 6 mo	6 mo	Dietary intake of SSBs or soft drinks 100% fruit juice, parental concern about the child’s diet	Dietary intake and attitude or intrinsic Motivation
Nollen et al [58], 2014, United States	N=51; age 9-14 y; n (CG)=25; n (IG)=26	To test the feasibility and potential efficacy of a 12-wk stand-alone mobile technology intervention for obesity prevention in girls aged 9-14 y	After-school program CG: no mobile technology IG: mobile technology	12 wk	BMI, dietary intake of vegetables, fruit, SSBs, or soft drinks	Anthropometric measurements and dietary intake
Nyström et al [31], 2018, Sweden; Nyström et al [33], 2017, Sweden	N=315;age 4 y; n (CG)=159 n (IG)=156	To assess the effectiveness of a mobile health (mHealth^f^) obesity prevention program on body fat, dietary habits, and physical activity in healthy Swedish children aged 4.5 y	Home CG: pamphlet on healthy eating and physical activity in preschool-aged children based on existing guidelines IG: smartphone app	6 mo	FMI^g^ (PO), FFMI^h^, dietary intake of vegetables, fruit, sugar-sweetened beverages or soft drinks, candy, composite score, physical activity, frequency of moderate-to-vigorous activity	Anthropometric measurements (PO), dietary intake, and physical activity
Pope et al [59], 2018, United States	N=105;age 16-18 y; n (CG)=not reported; n (IG)=not reported	To encourage high-school students to meet physical activity goals using a newly developed game and to document the feasibility, benefits, and challenges of using an electronic gaming app to promote physical activity in high school students	School CG: Fitbit (activity tracker) IG: Fitbit+Camp Conquer (activity tracker+electronic game)	12 wk	Physical activity (steps taken per day, active time per day)	Physical activity
Putnam et al [60], 2018, United States	N=131;age 4-5 y; n (CG)=44; n (IG1)=44 healthier condition; n (IG2)=43 unhealthy condition; n (IGT)=87	To examine whether children’s snack selections and consumption patterns are influenced by an app depicting a popular children’s media character, Dora the Explorer, as well as the role of children’s awareness of the character. The goal was to understand how to encourage healthier snack selection and consumption in newer game-based marketing venues, such as apps.	School CG: played a bowling game with no character IG1: played a bowling game with the character Dora with healthier snacks IG2: played a bowling game with the character Dora with unhealthy snacks	5 min of video+time to select and consume healthier or unhealthy snacks	Healthy food choices	Dietary intake
Røed et al [61], 2021, Norway	N=298;age 11 y; n (CG)=150; n (IG)=148	To examine the effect of a parent-focused eHealth intervention on children’s diet assessed at 2 time points postintervention. We hypothesized that, compared with the control group, the children in the intervention group would develop a more frequent and varied intake of fruits and vegetables and a less frequent intake of discretionary foods from baseline to postintervention.	Home CG: no use of website IG: use of website	6 mo	Dietary intake of vegetables, fruit, discretionary foods, variety of vegetable intake, variety of fruit intake	Dietary intake (PO was “Child diet quality” but no general results were reported. The PO was subdivided into secondary outcomes)
Rosi et al [62], 2016, Italy	N=58;age 8-10 y; n (CG)=33; n (IG)=58 (Master of Taste), 54 (Master of Taste supported by humanoid robot)	To evaluate whether the presence of a humanoid robot could improve the efficacy of a game-based, nutritional education intervention, to understand whether robot-child interactions can support nutritional learning in primary schools	School CG: no nutrition-oriented educational program IG1: exposed to a nutritional educational lesson (1 h per class) with both a theoretical and a playful phase, conducted by Master of Taste IG2: exposed to a nutritional educational lesson (1 h per class) with both a theoretical and a playful phase, conducted by Master of Taste with support from a humanoid robot	1 mo	Knowledge on nutrition	Knowledge
Sharma et al [63], 2015, United States	N=107;age 9-11 y; n (CG)=54; n (IG)=53	To evaluate the feasibility and acceptability of the Quest to Lava Mountain computer game and its effects on dietary behaviors, physical activity behaviors, and psychosocial factors among ethnically diverse children in Texas	School CG: continued with their usual programs IG: Quest to Lava Mountain was available to all children in the participating intervention classrooms	6 wk	Dietary intake of vegetables, fruit, carbohydrates, sugar or sucrose, fat, protein, fiber, calcium, total energy intake, and frequency of having breakfast. Physical activity: frequency of physical activity, frequency of outdoor physical activity, frequency of sport teams played, frequency of other physical activities, knowledge on nutrition and physical activity	Dietary intake, physical activity, and knowledge
Spook et al [64], 2016, Netherlands	N=453; age 16-21 y; n (CG)=225; n (IG)=228	To (1) examine the effects of BalanceIt on changes in (determinants of) secondary vocational education students’ dietary intake and physical activity, and (2) evaluate the uptake and use of the game and the game elements	School CG: no interventions were offered IG: participants received a link to the BalanceIt website and downloaded the BalanceIt app	4 wk	Dietary intake of vegetables, fruit, SSBs or soft drinks, physical activity, snacks or energy-dense snacks, fiber, frequency of moderate physical activity, frequency of vigorous physical activity, frequency of moderate-to-vigorous activity, attitude toward fruit and vegetables, snack and soft drink	Dietary intake, physical activity, attitude or intrinsic motivation (PO was “Dietary intake and physical activity” but no general results were reported. The PO was subdivided into secondary outcomes)
Thompson et al [32], 2016, United States; Thompson et al [34], 2015, United States	N=387;age 9-11 y; n (CG)=97; n (IG)=290 (98 action, 95 coping, and 97 both)	To evaluate research on a web-based serious video game (Squire’s Quest! II) that helps children eat more fruits and vegetables by using different strategies, such as planning when to eat them (action plans) or preparing for challenges (coping plans), and to see the short- and long-term effects of these strategies	School CG: played the game but only set a goal to eat fruits and vegetables, ie, did not create an action or coping implementation intention IG: (1) action group: set a fruit and vegetable goal and then created an action plan (ie, implementation intention); (2) coping group: set a goal to eat more fruits and vegetables then created a coping plan (ie, implementation intention); (3) both groups: set a goal to eat fruits and vegetables then created both action and coping plans	3 mo	Dietary intake of vegetables, fruit, and vegetables (PO)	Dietary intake (PO)
Wengreen et al [65], 2021, United States	N=1554;age 5-11 y; n (CG)=775; n (IG)=779	To assess the efficacy of the FIT game intervention in increasing vegetable consumption and skin carotenoid levels among schoolchildren and to evaluate the sustainability of these effects at 3-mo follow-up	School CG: did not receive any food education intervention IG: computer-based game designed to increase vegetable consumption	44 d in the first year and 39 d in the second year	Dietary intake of vegetables (PO), fruit, skin carotenoids concentration	Dietary intake (PO)
Wunsch et al [66], 2024, Germany	N=148 (74 adults, 74 children); age (mean): adults=47.8 y, children=13.3 y; n (CG)=64 (32 adults, 32 children); n (IG)=84 (42 adults, 42 children)	To evaluate the effectiveness of a theory-based mHealth intervention (SMARTFAMILY) in promoting physical activity and healthy eating in a collective family-based setting	Family-based setting CG: no intervention IG: participants used the SMARTFAMILY mHealth app to set family-based goals for physical activity, fruit and vegetable intake, and joint family meals. The app included goal setting, tracking, and weekly notifications for goal adjustments over a 3-wk period	3 wk	Dietary intake of fruit and vegetables. Physical activity (steps taken per day, frequency of moderate-to-vigorous activity); IPAQ^i^; 60 min screening measure (KIKA^j^)	Dietary intake and physical activity

^a^CG: control group.

^b^IG: intervention group.

^c^PO: primary outcome.

^d^DED-P: dietary energy density principle.

^e^SSB: sugar-sweetened beverage.

^f^mHealth: mobile health.

^g^FMI: fat mass index.

^h^FFMI: fat-free mass index.

^i^IPAQ: International Physical Activity Questionnaire.

^j^KIKA: 60-Minute Screening Measure.

**Table 2:** Description of the interventions.

Study, year	Intervention characteristics	Digital tool	Digital and other components’ uses
Bannon and Schwartz [35], 2006	Nutrition message framing videos	Videos	Videos used to influence snack choices among kindergartners through gain-framed and loss-framed messages
Baños et al [36], 2013	The Fantastic Food Challenge game and ETIOBE Mates website	Computer game and website	A game and website designed to teach children various aspects of nutrition knowledge in an engaging way
Baranowski et al [37], 2003; Cullen et al [38], 2005	Squire’s Quest! multimedia game	Multimedia game	A psychoeducational game to increase preferences for fruits, juice, and vegetables through multiple exposures and associating their consumption with fun
Baranowski et al [39], 2011	Escape from Diab and Nanoswarm video games	Video games	Evaluates the effects on children’s diet, physical activity, and adiposity through engaging and educational gameplay
Byrne et al [40], 2012	Mobile phone game	Smartphone with virtual pet game	Encourages adolescents to eat breakfast by interacting with a virtual pet that responds to their breakfast behaviors
Carlin et al [41], 2021	Intelligent personal assistant	Smart speaker (Echo Dot)	Prompts and reminders related to public health recommendations in relation to physical activity and dietary habits; voluntary prompts related to topics of interest such as health and fitness, lifestyle, sport, cooking, and recipes
Chagas et al [42], 2020	Nutritional intervention using a digital game	Digital game	Aims to have an impact on food consumption, nutrition knowledge, and healthy eating practices among high-school students
Clarke et al [43], 2019	VeggieBook mobile app	Smartphone app	Offers personalized recipes and tips for vegetable-based meals and snacks, with interactive and social sharing features
de Vlieger et al [44], 2021	VitaVillage nutrition education game	Game (Unity3D)	An educational game designed to improve children’s nutrition knowledge through interactive gameplay and educational content
Espinosa-Curiel et al [22], 2020	Promotion of healthy behaviors via serious game sessions	Serious game (HelperFriend)	Kinect-based game, providing feedback to improve children’s healthy behavior choices
Fassnacht et al [45], 2015	SMS text messaging-based monitoring and feedback for health behaviors	SMS text messaging-based system	Children self-reported health behaviors via SMS text messaging and received feedback to encourage behavior change
Folkvord et al [46], 2013	Advergames promoting snacks or fruit	Advergames	Examines the influence of advergames on children’s snack and fruit consumption
Folkvord et al [47], 2021	Garfield versus hot dog serious game	Serious game	Uses behavioral change techniques within a game to improve eating behavior among children
Froome et al [48], 2020	Foodbot Factory mobile app	Game mobile app	A pilot study to improve children’s knowledge of Canada’s food guide through interactive modules and facilitated sessions
Gan et al [49], 2019	Healthy Foodie nutrition game app	Digital card game (Rango Cards)	A randomized controlled trial to determine the effectiveness of the game on children’s nutrition knowledge
Haddad et al [50], 2023	Children involved in cooking at home using a smartphone app	Smartphone app	The app provided recipes and tracked children’s intake and preferences for wholemeal pasta and brussels sprouts. Parents reported children’s meal liking and intake using the app
Hammersley et al [51], 2019	Internet-based program for parents focusing on healthy lifestyles for preschool-aged children	Internet-based program	The program included modules on nutrition, physical activity, and sleep, with individual feedback from a dietitian and a closed Facebook group for support
Heikkilä et al [52], 2019	Mobile app for nutrition education	Mobile app	A mobile app used in conjunction with participatory nutrition education sessions to improve nutrition knowledge and dietary intake
Hermans et al [53], 2018	Alien Health Game and Super Shopper game	Video game	A game designed to teach nutrition and healthy food choices to elementary schoolchildren
Kato-Lin et al [54], 2020	Fooya! mobile app game	Mobile app game	A game that encourages children to maintain a healthy diet and exercise through gameplay that affects the avatar’s body shape and abilities
Mack et al [55], 2020	Motion-controlled serious game	A game with physical interaction	A game that promotes knowledge about nutrition and a healthy lifestyle through active player movement and task completion
Marsh et al [56], 2015	Screen-based intervention	Multimedia setup	An experimental setup to assess the impact of single versus multiple screen access on eating behavior in adolescents
Nezami et al [57], 2018	Maternal-targeted intervention to reduce children’s sugar-sweetened beverage intake	Mobile-optimized website and text messages	Mothers received weekly lessons, tracked daily beverage intake via text, and received tailored feedback via email to promote self-regulation and behavior change
Nollen et al [58], 2014	Mobile technology intervention	Handheld computer device (MyPal A626)	Stand-alone intervention targeting fruits or vegetables, sugar-sweetened beverages, and screen time through goal setting, planning, cues to action, self-monitoring, and feedback
Nyström et al [31], 2018; Nyström et al [33], 2017	MINISTOP smartphone app	Smartphone app	An mHealth^a^ obesity prevention program delivered via a smartphone app to parents, focusing on healthy eating and physical activity for preschool-aged children
Pope et al [59], 2018	Camp Conquer gaming intervention	Gaming app	A physical activity promotion game where students’ activity levels affect gameplay outcomes
Putnam et al [60], 2018	Dora with healthier products intervention	App game	Influences snack selections and consumption patterns through an app featuring Dora the Explorer with healthier snacks
Røed et al [61], 2021	Food4toddlers eHealth intervention	Website	Provides modules, recipes, and a forum focused on promoting healthy food environments for children
Rosi et al [62], 2016	Giocampus educational game	Educational game	Uses a playful approach to teach nutritional concepts and healthy eating practices to children
Sharma et al [63], 2015	Quest to Lava Mountain computer game	Web-based computer game	A game based on social cognitive theory to improve dietary and physical activity behaviors among children
Spook et al [64], 2016	Serious self-regulation game (BalanceIt)	Interactive multimedia game	A game designed to influence dietary intake and physical activity through self-regulation techniques and a peer support system
Thompson et al [32], 2016; Thompson et al [34], 2015	Squire’s Quest! II video game	Web-based video game	Aims to encourage children to consume fruits and vegetables through engaging gameplay and behavioral change techniques
Wengreen et al [65], 2021	FIT game intervention	Digital episodes displayed on screen	A series of comic-book formatted episodes shown in the school cafeteria to promote vegetable consumption among children
Wunsch et al [66], 2024	Family-based mHealth intervention promoting physical activity and healthy eating	SMARTFAMILY app	The app provided goal setting, self-monitoring, feedback, and social support through behavioral change techniques for family members

^a^mHealth: mobile health.

Of 34 studies, 5 (15%) focused on young children aged 2 to 5 years [31,33,35,51,57,60], while 20 (59%) targeted primary school-aged children between 6 and 12 years [12,22,30,32,34-39,41-44,46,48-50,53,55,62,65].

Of 34 studies, 2 (6%) focused on children aged 9 to 14 [43,58] and 7 (21%) were conducted among adolescents aged 13 to 19 years [32,39,40,42,43,56,59,63,64,66,67]. In 2 (6%) studies, young adults aged 16 to 21 years were included [52,64]. Family-based interventions, where both children and parents participated, were included in 2 (6%) studies [41,66].

Of 34 studies, 20 (59%) implemented school-based interventions, while home-based interventions were used in 8 (24%) studies [31,33,41,50-52,54,57,61]. In total, 2 (6%) studies used mixed settings, integrating both school and home components [48,55]. The remaining 4 (12%) studies were conducted in a food pantry [41], a study clinic [56], or an after-school program [58].

Of 34 studies, 9 (26%) exclusively targeted dietary intake, such as fruit and vegetable intake or healthy food choices [32,34,35,37,38,43,54,56,60,61,65]. In total, 8 (24%) studies exclusively targeted nutritional knowledge [22,36,42,44,48,49,62,63]. In total, 2 (6%) studies assessed only physical activity levels [41,59]. A total of 4 (12%) studies focused on both dietary intake and physical activity [39,45,46,66]. In total, 3 (1%) studies focused on dietary intake and knowledge [52,53,55], with 1 (3%) of these also including physical activity [55]. In addition, 5 (15%) studies incorporated other outcomes, such as attitudes or intrinsic motivation [40,47,50,57,64].

Anthropometric measurements were included in 9% (3/34) studies [31,33,51,58], while 3% (1/34) study included 4 outcome types: anthropometric measurements, dietary intake, physical activity, and attitude or intrinsic motivation [51].

Across the 34 studies, a primary outcome was clearly identified in 9 (26%; Multimedia Appendix 5) studies. These included outcomes were dietary intake of fruit, juice, and vegetables [37]; dietary intake of target vegetable preparations [43]; dietary intake of vegetables, fruit, or fruit and vegetables [32,34,65]; total energy intake [56]; knowledge of nutrition [48,52]; BMI [51]; and fat mass index [31,33]. Altogether, in 65% (22/34) of studies, it was unclear what the primary outcome was. In 53% (18/34) of those, there was no sample size calculation [22,35,36,40-42,44-47,50,53,57-60,62,63], and in 12% (4/34) studies, it was not clear for what outcome the sample size was calculated [39,49,54,66]. In total, 9% (3/34) of studies were referred to as pilot studies without a power calculation [22,41,58]. The status of the outcomes (primary, secondary, and unclear) is marked in Multimedia Appendix 5 and as relevant in Table 3.

**Table 3:** A summary of intervention effects (in comparison to the control group) found in the included studies for anthropometric measurements, dietary habits, physical activity, knowledge, and attitude outcomes. The results are only shown for the outcomes that were evaluated by at least 2 studies.

	Improvement in outcome	No change in outcome
BMI (kg/m^2^)	—^a^	3^b^
Vegetables	2	8
Fruit	5	6
Fruits and vegetables	2	1
Sugar-sweetened beverage	2	2
Candy	—	1
Banana	—	2
Carbohydrates	—	2
Sugar or sucrose	1	2
Fat	—	2
Protein	—	2
Total energy intake	1	3^b^
Healthy food choices	2	1
Active time per day	—	2
Sedentary time per day	—	3
Frequency of light activity	—	2
Frequency of moderate physical activity	—	2
Frequency of vigorous physical activity	—	2
Frequency of moderate-to-vigorous activity	—	3
Attitude or intrinsic motivation knowledge	—	—
Nutrition	4^b^	2^b^

^a^Not available.

^b^The reported outcome was the primary outcome in one of the studies. All other outcomes reported in the table were either secondary outcomes or the status was unclear.

Among the included RCTs, 35% (12/34; reported in 14 articles) were from the United States [32,34,35,37-40,43,57-60,63,65]. The European studies were from Spain [36], the United Kingdom [41], Italy [62], Sweden [31,33], Norway [61], the Netherlands [47,53,64], Germany [45,55,66], Finland [52], and Switzerland [50]. Studies from other continents included Brazil [42], Australia [44,51], New Zealand [56], India [54], the Philippines [49], Canada [48]*,* and Mexico [22].

### Effectiveness of the Interventions

Multimedia Appendix 5 shows the effects of all the interventions on all the various outcomes, and Table 3 summarizes the main findings.

#### Dietary Intake

Most positive results were observed for fruit intake: 5 of 11 (45%) studies succeeded in increasing intake, from which the effect was ascertained with an objective measure (skin carotenoid concentration) [65]. In total, 4 of 11 (36%) studies targeting the intake of fruit and vegetables have succeeded only in increasing fruit intake [32,34,37-39,65], which could indicate greater acceptability of fruits than vegetables among children. Nevertheless, 9% (1/11) of studies succeeded in increasing vegetable intake, but not fruit intake [61].

The most common behavioral change techniques (BCTs) were goal setting, instructions on how to perform a behavior, self-monitoring of behavior, and action planning (Multimedia Appendix 6). Only 33% (2/6) studies using feedback on behavior were successful [4,45]. However, all of them used a different combination of BCTs, in which there was only one feedback, and no clear pattern of BCTs could be seen in the successful versus unsuccessful intervention.

The 2 (50%) successful interventions out of a total of 4 interventions with the aim of decreasing sugar-sweetened beverage (SSB) use were nongame mobile apps [33,57]. They both included a mobile app for use by the parents of 4-year-old children [33] or preschool-aged children [57]. The main difference compared with the other interventions reporting SSB intake [6,32] was that they targeted much younger children (4 years and preschool age vs 9-11 years) and the app was used by the parents instead of the children. The primary aims of the interventions also differed—the studies by Nyström et al [33] and Nollen et al [58] were obesity prevention interventions, and Thompson et al [32] primarily targeted increased fruit and vegetable consumption, while the study by Nezami et al [57] targeted decreased SSB intake. A computer game intervention aiming to increase fruit, vegetables, and whole-grain intake and to decrease added sugar and fat intake succeeded in decreasing sugar intake but not in changing the other intakes. Given the small number of different types of studies, it is difficult to make generalized conclusions about what types of interventions would be successful in decreasing sugar intake in children.

Out of 34 studies, 4 (12%) studies have reported changes in the consumption of snacks, candy, or chocolate [31-33,46], of which 1 (25%) was successful [32], 1 (25%) had negative effects [46], and the rest had no effect. The successful intervention applied goal setting and planning of how to meet the goal, in addition to a game as the intervention. The one with negative effects used an advergame (a free web-based game integrating advertising messages, logos, and trade characteristics) as an intervention. Interestingly, cues of healthy and unhealthy foods in the advergame resulted in increased intake of unhealthy foods.

The persistence of increased fruit intake and carotenoid concentrations 3 months after the end of the FIT game intervention [65] and 3 months after the end of the game and action plan intervention in the studies by Thompson et al [32,34], and reduced discretionary food intake 6 months after the end of the eHealth intervention Time2bHealthy [51], contradicts the general pattern reported by others [61]. The lack of sustained results in the study by Nyström et al [31] aligns with this conclusion. The longer follow-up period (12 months) in the study by Nyström et al [31] could explain the difference in the nonpersistence of changed food intake compared with that in Thompson et al [32], Wengreen et al [51], and Hammersley et al [65].

#### Anthropometric Measurements

Regarding anthropometric measurements, of 34 studies, 1 (3%) study revealed an effect on fat-free mass [31,33]. The lack of effect in the other studies may be attributed to the fact that, on average, the study participants were not overweight [6,33] or the intervention was too short to have measurable results (12 weeks in the study by Nollen et al [58], <2 months in the study by Baranowski et al [39]). In addition, only the studies by Nyström et al [33] and Hammersley et al [51] reported that a sample size calculation was based on the chosen anthropometric outcome, whereas the study by Nollen et al [58] did not perform a power calculation, and the study by Baranowski et al [39] did not mention for what variable the sample size calculation was performed.

#### Physical Activity

Overall, the articles that evaluated any aspect associated with physical activity indicated no effect of the intervention [6,33,45,63,66]; of 34 studies, 1 (3%) reported that the sample size was too small [41], and the other had an attrition rate too large for statistical analysis [59].

#### Knowledge and Attitudes

In total, 2 (50%) out of 4 game-based studies reported beneficial effects on attitudes toward healthy eating or nutrition, and physical activity [40,52]. Both studies were rather small, which limits the interpretability of the results. Most of the studies reporting on knowledge about nutrition, food, or physical activity were successful. Interestingly, studies with no impact targeted older individuals—13 to 19 years [42] and 18 years [57]—compared with successful studies (7-13 years). In the study by Chagas et al [42], the control group did not receive any intervention. In the study by Heikkilä et al [52], the control group also participated in nutrition education sessions but without a mobile app intervention. Both the intervention and control groups increased their nutrition knowledge, but there was no difference between the groups, suggesting no additional benefit from the mobile app.

### Risk-of-Bias Assessment

Table 4 shows the risk-of-bias assessment for each study. The risk of bias due to random sequence generation was considered low for all 34 included studies (37 publications) [22,31-44,46,48-51,53,55-60,62-67]. Concerns of either high or unclear risk of bias were raised in all other domains for some studies, ie, allocation concealment (17/34, 50% studies); blinding of participants and personnel (25/34, 74% studies); blinding of outcome assessment (28/34, 82% studies); incomplete outcome data (18/34, 53% studies); selective outcome reporting (3/34, 9% studies); and other biases (16/34, 47% studies). The interventions conducted in the studies by Nollen et al [58] and Thompson et al [34] were considered to have a low risk of bias in all domains, and those conducted by Folkvord et al [46] and Kato-Lin et al [54] were considered to have a low risk of bias in all but one domain.

**Table 4:** Risk of bias for the included studies.

Study, year	Random sequence generation (selection bias)	Allocation concealment (selection bias)	Blinding of participants and personnel (performance bias)	Blinding of outcome assessment	Incomplete outcome data (attribution bias)	Selective reporting (reporting bias)	Other sources of bias
Bannon and Schwartz [35], 2006	+^a^	–^b^	×^c^	×	+	×	+
Baños [36], 2013	+	–	–	–	×	+	+
Baranowski et al [37], 2003	+	–	–	–	+	+	+
Baranowski et al [39], 2011	+	–	+	–	+	+	+
Byrne et al [40], 2012	+	–	–	–	–	–	+
Carlin et al [41], 2021	+	+	×	–	+	+	×
Chagas et al [42], 2020	+	+	×	–	×	+	–
Clarke et al [43], 2019	+	+	+	–	–	–	×
Cullen et al [38], 2005	+	–	–	–	+	+	+
de Vlieger et al [44], 2022	+	–	–	–	×	+	×
Espinosa-Curiel et al [22], 2020	+	+	–	–	+	–	–
Fassnacht et al [45], 2015	+	–	×	–	–	+	–
Folkvord et al [46], 2013	+	+	+	–	+	+	+
Folkvord et al [47], 2021	+	×	×	×	×	+	×
Froome et al [48], 2020	+	×	+	+	×	+	+
Haddad et al [50], 2023	+	+	–	–	–	+	–
Hammersley et al [51], 2019	+	+	–	+	–	+	–
Heikkilä et al [52], 2019	+	×	–	×	×	+	+
Hermans et al [53], 2018	+	+	+	–	+	+	–
Kato-Lin et al [54], 2020	+	+	+	+	–	+	+
Mack et al [55], 2020	+	+	×	×	+	+	+
Marsh et al [56], 2015	+	+	×	–	+	+	×
Nezami et al [57], 2018	+	+	–	–	–	+	–
Nollen et al [58], 2014	+	+	+	+	+	+	+
Nyström et al [33], 2017	+	+	×	+	+	+	+
Nyström et al [33], 2018	+	+	×	+	+	+	+
Pope et al [59], 2018	+	–	–	–	×	+	–
Putnam et al [60], 2018	+	–	–	–	+	+	–
Gan et al [49], 2019	+	–	–	–	+	+	+
Røed et al [61], 2021	+	+	–	–	×	+	+
Rosi et al [62], 2016	+	–	–	–	+	+	+
Sharma et al [63], 2015	+	–	–	–	+	+	+
Spook et al [64], 2016	+	+	–	–	×	+	×
Thompson et al [34], 2015	+	+	+	+	+	+	+
Thompson et al [32], 2016	+	+	+	+	+	+	+
Wengreen et al [65], 2021	+	–	+	–	+	+	+
Wunsch et al [66], 2024	+	+	–	+	–	+	–

^a^Low risk.

^b^Unclear.

^c^High risk.

## Discussion

### Dietary Intake

The interventions showed variable effects on dietary intake—many of the dietary outcomes related to food and energy intake remained unaltered by the interventions conducted. However, the reported outcomes were not always the main targets of the intervention, which probably explains some of the null results. Most positive results were observed for fruit intake, from which all but one used games as an intervention. The more frequent success in fruits compared with vegetables could indicate greater acceptability of fruits than vegetables among children. Other SRs have also shown that the most reported successful effect on dietary intake is fruit consumption [8,27,43,62]. Our findings also suggest that targeting parents instead of children may be more effective in reducing SSB consumption in children under school age.

This result is in accordance with previous studies targeting parents with educational content to reduce children’s SSB intake [68]. The negative implication of increased energy-dense food consumption after playing advergames, regardless of the type of advertised food (energy-dense foods or fruits) [46], calls for more research on the psychological mechanisms of games.

In our study, both maintained and not maintained long-term effects on fruit and vegetable intake were seen [31,32,34,65]. However, the general pattern reported by others seems to be a lack of long-term effects [61]. It may be that the use of digital tools needs to be recurrent to achieve long-term effects. In the future, it will be important to study the long-term effects of different digital tool interventions. Regarding other dietary behaviors, it is impossible to draw conclusions because of the small number of studies.

### Anthropometric Measurements

It is difficult to draw firm conclusions about the potential of mobile apps or games to improve anthropometric measurements because of the shortcomings of the studies. However, the lack of effect found in this review is in accordance with other SRs in which no changes were found regarding anthropometric outcomes in children and adolescents [8,69]. Future studies should target overweight instead of normal weight children, be long enough to have an impact, and apply a sample size calculation.

### Physical Activity

In this SR, studies involving physical activity interventions without dietary intervention were excluded, and our review focused only on combined dietary or physical activity interventions. This probably explains the differences compared with other SRs, which have also included physical activity interventions without dietary interventions [27,63,69]. Therefore, our results provide a limited view of the potential of digital tools for assessing children’s physical activity.

### Knowledge and Attitudes

The results suggest that the interventions were successful in increasing young children’s knowledge about food and nutrition. Regarding attitudes, the small number of studies does not allow firm conclusions. In addition, the use of different types of control groups between interventions complicated comparisons of the effectiveness of the studies. Inconclusive results regarding adolescents’ knowledge, attitudes, and skills were also found in other reviews focused on the use of digital interventions [62].

### Behavior Change Theories and Techniques

In several articles, researchers referred to psychological theories relevant to the creation of interventions aimed at improving health behaviors and attitudes in children. Most authors referred to well-known psychological concepts, such as social cognitive theory [31,37,39,42,61] and self-determination theory [52,65]. However, mentioning a theory provided little insight into what BCTs the theory led to. Describing the actual BCTs, as was done by many studies, was more informative. We compared the effectiveness of different BCTs by categorizing them based on the BCT taxonomy by Michie et al [70]. However, no clear pattern was seen. This could have been affected by (1) too different combinations of BCTs between the studies, (2) all interventions using the same core BCTs, making little difference between the interventions, or (3) us not capturing all BCTs in some interventions because of a lack of detailed description. While it is worth considering incorporating psychological concepts and well-founded theoretical frameworks into the design and development of interventions [47], a detailed enough description of the BCTs is necessary for appropriate comparison of successful and unsuccessful BCTs.

Some of the games were linked with changing real-life behaviors, for example, through setting goals for behaviors outside the game environment. Others included goal setting and behavior change only in the game environment. Although this could be meaningful regarding the success of the intervention, the frequency of these was not different between the successful and unsuccessful interventions.

### Strengths and Limitations of Our SR

This review has several limitations that must be considered when interpreting the findings.

First, it is challenging to draw conclusions based on studies with substantial heterogeneity in intervention type, duration, and control group comparisons. For example, the appeal and engagement of the digital tools may have differed significantly across studies, but compliance data were not consistently reported.

Second, many studies lacked rigorous sample size justification: 18 (53%) out of 34 studies did not report a power calculation, and among those that did, several did not clarify the outcome for which the calculation was performed. This may have resulted in underpowered analyses and limited detection of statistically significant effects.

Third, we did not conduct hand-searching or include gray literature, which may have excluded potentially relevant but unindexed or unpublished studies. This could have reinforced the effect of publication bias in our synthesis.

Fourth, the use of single reviewers for most of the title or abstract and full-text screening stages is a methodological limitation. Although only 20% of the records were screened in duplicate, the interrater agreement (using Cohen κ) was “almost perfect” for title or abstract screening and “strong” for full-text eligibility assessment [71].

Fifth, we acknowledge that categorizing findings by outcome rather than by intervention type or study setting may limit insights into which characteristics of digital tools were most influential in promoting behavior change. Future reviews may benefit from structuring the synthesis accordingly. Although we mapped the BCTs used in the studies investigating the effect on fruit and vegetable intake, the variety in combinations of BCTs among the studies prevented us from drawing conclusions.

Sixth, we were unable to perform a Grading of Recommendations, Assessment, Development and Evaluations assessment due to limited resources. Although we assessed the risk of bias in detail, this precluded a formal evaluation of the certainty of the evidence, which would have further strengthened the interpretability of the findings.

Seventh, we did not conduct a meta-analysis due to the high degree of heterogeneity across interventions, outcomes, and measurement tools. As such, we are unable to comment on the pooled effect size or clinical significance of the interventions studied.

Eighth, additional subgroup and sensitivity analyses—such as stratification by age group or publication year—may have provided further insights. The inclusion of older studies, while justified by our broad inclusion criteria, may limit the relevance of some findings, given the rapid evolution of digital technologies. Despite these limitations, some of the strengths of this review are a thorough examination of the quality of the studies and the use of a preregistered protocol.

### Recommendations

Given the findings from our SR, which highlighted the variable effectiveness of mobile- and web-based interventions in improving health behaviors among children and adolescents, the following recommendations are proposed to enhance the design and evaluation of future interventions:

- Explore the differences in the features of different tools and the role of the features in the effectiveness of the tools. It is crucial to extend the follow-up periods in future studies and compare different durations of the use of the same tool to assess the sustainability of behavioral changes. Research should aim to measure long-term outcomes beyond the immediate postintervention phase to better understand the lasting impact of these interventions.
- Explore the role of parental involvement and family dynamics in the success of interventions, given their significant influence on children’s health behaviors.
- Investigate the scalability and real-world applicability of successful interventions, including assessments of cost-effectiveness and barriers to implementation in diverse settings.
- Enhance the methodological quality of research by ensuring adequate sample sizes and blinding where feasible to strengthen the evidence base supporting these interventions.
- Enhance the theoretical underpinnings of interventions by applying behavior change theories more systematically. Future research should clearly outline how behavior change theories are integrated into intervention design and evaluation.

### Conclusions

Our SR explored the impact of mobile- and web-based interventions on dietary habits, physical activity, and obesity-related health outcomes in children and adolescents. Despite the diverse array of interventions examined, the results illustrated a complex efficacy landscape. We found that while some interventions, particularly games, showed promise in improving short-term dietary behaviors, notably increasing fruit and vegetable intake, their impact on long-term health outcomes and changes in behavior, such as reducing sedentary time, was inconclusive.

The effectiveness of these interventions varied, with a notable number of studies showing improved nutrition knowledge and some reporting positive shifts in dietary behaviors. However, in the few long-term follow-ups, the changes were not always sustained over time, highlighting the challenges of maintaining health behavior changes initiated through digital interventions.

Given the mixed outcomes, it is important to further investigate the specific features of the tools that are effective. Future studies should focus on developing interventions that not only engage children and adolescents effectively but also include components that support sustained health behaviors.

## Appendix

Multimedia Appendix 1 Search strategies.

Multimedia Appendix 2 Query results per database.

Multimedia Appendix 3 Inclusion and exclusion criteria.

Multimedia Appendix 4 Data collection form.

Multimedia Appendix 5 Outcomes.

Multimedia Appendix 6 Behavior change techniques.

Multimedia Appendix 7 PRISMA (Preferred Reporting Items for Systematic Reviews and Meta-Analyses) checklist.

## Abbreviations

BCT: behavioral change technique
PICOST: population, intervention, comparison, outcomes, study design, and time
PRISMA: Preferred Reporting Items for Systematic Reviews and Meta-Analyses
RCT: randomized controlled trial
SR: systematic review
SSB: sugar-sweetened beverage

## Data Availability

The datasets generated or analyzed during this study are available from the corresponding author on reasonable request.
