# Supplementary material for: Mobile- and Web-Based Interventions for Promoting Healthy Diets, Preventing Obesity, and Improving Health Behaviors in Children and Adolescents: Systematic Review of Randomized Controlled Trials
Source: J Med Internet Res. 2025 May 20;27:e60602. doi: 10.2196/60602 (PMC12134700; doi:10.2196/60602)
Supplement: Multimedia Appendix 1 [file jmir_v27i1e60602_app1.docx]

**Multimedia Appendix 1: Keywords, databases and search strings used in the study**

| **Keywords** | adolescents; allforkids; anthropometric; attitudes; behavior; bmi; children; clinical; comparison; consumption; dietary; diet; digital; ehealth; effectiveness; family; food; gamification; games; health; healthy; intake; interventions; kids; knowledge; lifestyle; meal; mobile; nutrition; outcomes; physical; public health; sedentary; tools; weight |
| --- | --- |
| **Database** | **Search strategy** |
| PubMed | (((("Children knowledge" OR children OR knowledge OR adolescents OR kids OR allforkids OR family) AND ("games for health" OR "health game" OR "Mobile phone" OR "digital tools" OR "gamification")) AND ("Dietary habits" OR "food habits" OR "dietary choices" OR "healthy eating" OR "healthy lifestyle" OR eating behavior OR ehealth)) AND (food)) AND (Nutrition OR "nutrient intake" OR "food consumption" OR meal OR "public health" OR "dietary guidelines")  https://pubmed.ncbi.nlm.nih.gov/?term=%28%28%28%28%22Children+knowledge%22+OR+children+OR+knowledge+OR+adolescents+OR+kids+OR+allforkids+OR+family%29+AND+%28%22games+for+health%22+OR+%22health+game%22+OR+%22Mobile+phone%22+OR+%22digital+tools%22+OR+%22gamification%22%29%29+AND+%28%22Dietary+habits%22+OR+%22food+habits%22+OR+%22dietary+choices%22+OR+%22healthy+eating%22+OR+%22healthy+lifestyle%22+OR+eating+behavior+OR+ehealth%29%29+AND+%28food%29%29+AND+%28Nutrition+OR+%22nutrient+intake%22+OR+%22food+consumption%22+OR+meal+OR+%22public+health%22+OR+%22dietary+guidelines%22%29 |
| Web of Science | **TS=("Children knowledge" OR children OR knowledge OR adolescents OR kids OR allforkids OR family)** **AND** **TS=("games for health" OR "health game" OR "Mobile phone" OR "digital tools" OR gamification)** **AND** **TS=("Dietary habits" OR "food habits" OR "dietary choices" OR "healthy eating" OR "healthy lifestyle" OR "eating behavior" OR ehealth)** **AND** **TS=(food)** **AND** **TS=(Nutrition OR "nutrient intake" OR "food consumption" OR meal OR "public health" OR "dietary guidelines")**  https://www.webofscience.com/wos/woscc/summary/47dd0f61-311f-467d-a238-4ad5f186e8b8-010cb00d31/relevance/1 |
| Scopus | TITLE-ABS-KEY ( "Children knowledge" OR children OR knowledge OR adolescents OR kids OR allforkids OR family ) AND TITLE-ABS-KEY ( "games for health" OR "health game" OR "Mobile phone" OR "digital tools" OR gamification ) AND TITLE-ABS-KEY ( "Dietary habits" OR "food habits" OR "dietary choices" OR "healthy eating" OR "healthy lifestyle" OR ehealth ) AND TITLE-ABS-KEY ( food ) AND TITLE-ABS-KEY ( nutrition OR "nutrient intake" OR "food consumption" OR meal OR "public health" OR "dietary guidelines" ) |
| Google Scholar | Children knowledge OR children OR knowledge OR adolescents OR kids OR allforkids OR family OR games for health OR health game OR Mobile phone OR digital tools OR gamification OR Diet |
