# Supplementary material for: Mobile- and Web-Based Interventions for Promoting Healthy Diets, Preventing Obesity, and Improving Health Behaviors in Children and Adolescents: Systematic Review of Randomized Controlled Trials
Source: J Med Internet Res. 2025 May 20;27:e60602. doi: 10.2196/60602 (PMC12134700; doi:10.2196/60602)
Supplement: Multimedia Appendix 3 [file jmir_v27i1e60602_app3.docx]

**Multimedia Appendix 3: Inclusion and exclusion criteria**

| **Criterion** | **Inclusion Criteria** | **Exclusion Criteria** |
| --- | --- | --- |
| **Population** | Children and adolescents aged 6 months to 18 years | Studies with adult participants (>18 years) or populations outside the specified age range. |
| **Intervention** | Mobile-based, web-based, or game-based interventions promoting healthy diets, physical activity, or both. | Interventions not delivered through mobile, web, or digital platforms; interventions not related to health. |
| **Comparison** | Comparisons with traditional interventions, no intervention, or other digital interventions. | Studies without a comparison group or that compare irrelevant interventions (e.g., pharmaceutical only). |
| **Outcomes** | Primary outcomes related to dietary habits, physical activity, or changes in nutrition knowledge or behavior. | Studies not measuring relevant health outcomes (e.g., outcomes unrelated to nutrition or physical activity). |
| **Study Design** | Randomized controlled trials (RCTs), controlled before-after studies, and cohort studies. | Cross-sectional studies, case reports, non-controlled studies, or qualitative studies. |
| **Publication Type** | Peer-reviewed articles, conference proceedings, and theses available in full text. | Abstracts only, non-peer-reviewed studies, and protocols without results. |
| **Language** | Studies published in English, Spanish, Finnish, and Polish. | Studies published in languages other than those specified. |
| **Setting** | Studies conducted in school, community, or home settings involving digital health interventions. | Studies conducted in clinical or hospital settings unless they focus specifically on digital interventions. |
