# Supplementary material for: Mobile- and Web-Based Interventions for Promoting Healthy Diets, Preventing Obesity, and Improving Health Behaviors in Children and Adolescents: Systematic Review of Randomized Controlled Trials
Source: J Med Internet Res. 2025 May 20;27:e60602. doi: 10.2196/60602 (PMC12134700; doi:10.2196/60602)
Supplement: Multimedia Appendix 4 [file jmir_v27i1e60602_app4.docx]

**Multimedia Appendix 4:**
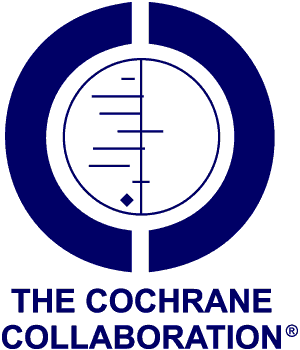
**Data collection form**

Intervention review – RCTs only

This form can be used as a guide for developing your own data extraction form. Sections can be expanded and added, and irrelevant sections can be removed. It is difficult to design a single form that meets the needs of all reviews, so it is important to consider carefully the information you need to collect, and design your form accordingly. Information included on this form should be comprehensive, and may be used in the text of your review, ‘Characteristics of included studies’ table, risk of bias assessment, and statistical analysis.

## Notes on using a data extraction form:

- Be consistent in the order and style you use to describe the information for each report.
- Record any missing information as unclear or not described, to make it clear that the information was not found in the study report(s), not that you forgot to extract it.
- Include any instructions and decision rules on the data collection form, or in an accompanying document. It is important to practice using the form and give training to any other authors using the form.

| Study title or ID |  |
| --- | --- |
| Study ID *(surname of first author and year first full report of study was published e.g. Smith 2001)* |  |
| Notes | |

# General Information

| Date form completed *(dd/mm/yyyy)* |  |
| --- | --- |
| Name/ID of person extracting data |  |
| Reference citation |  |
| Study author contact details |  |
| Publication type  *(e.g. full report, abstract, letter)* |  |
| Notes: | |

# Study eligibility

| Study Characteristics | Eligibility criteria  *(Insert inclusion criteria for each characteristic as defined in the Protocol)* | | Eligibility criteria met? | | | Location in text or source *(pg & ¶/fig/table/other)* |
| --- | --- | --- | --- | --- | --- | --- |
|  |  | | Yes | No | Unclear |  |
| Type of study |  | |  |  |  |  |
|  |  | |  |  |  |  |
| Participants  (age 18y. or younger) |  | |  |  |  |  |
| Types of intervention |  | |  |  |  |  |
| Types of comparison  Traditional food education / other food education /no food education |  | |  |  |  |  |
| Types of outcome measures  Food acceptance, changes in food behaviour, food consumption... |  | |  |  |  |  |
| INCLUDE | | EXCLUDE | | | | |
| Reason for exclusion |  | | | | | |
| Notes: | | | | | | |

**DO NOT PROCEED IF STUDY EXCLUDED FROM REVIEW**

# Characteristics of included studies

## Methods

|  | **Descriptions as stated in report/paper** | | **Location in text or source** *(pg & ¶/fig/table/other)* |
| --- | --- | --- | --- |
| **Aim of study** *(e.g. efficacy, equivalence, pragmatic)* |  | |  |
| **Design***(e.g. parallel, crossover, non-RCT)* |  | |  |
| **Unit of allocation**  *(by individuals, cluster/ groups or body parts)* |  | |  |
| **Start date** |  | |  |
| **End date** |  | |  |
| **Duration of participation**  *(from recruitment to last follow-up)* |  | |  |
| **Ethical approval needed/ obtained for study** | Yes No Unclear |  |  |
| **Notes:** | | | |

## Participants

|  | Description  *Include comparative information for each intervention or comparison group if available* | | Location in text or source *(pg & ¶/fig/table/other)* |
| --- | --- | --- | --- |
| Population description  *(from which study participants are drawn)* |  | |  |
| Setting  *(including location and social context)* |  | |  |
| Inclusion criteria |  | |  |
| Exclusion criteria |  | |  |
| Method of recruitment of participants *(e.g. phone, mail, clinic patients)* |  | |  |
| Informed consent obtained | Yes No Unclear |  |  |
| Total no. randomised |  | |  |
| Clusters  *(if applicable, no., type, no. people per cluster)* |  | |  |
| Baseline imbalances |  | |  |
| Withdrawals and exclusions  *(if not provided below by outcome)* |  | |  |
| Age |  | |  |
| Sex |  | |  |
| Race/Ethnicity |  | |  |
| Other relevant sociodemographics  Education, Geographic residence, BMI, Family type/size, Language |  | |  |
| Subgroups measured |  | |  |
| Subgroups reported |  | |  |
| Notes: | | | |

## Intervention groups

*Copy and paste table for each intervention and comparison group*

**Intervention Group 1**

|  | Description as stated in report/paper | Location in text or source *(pg & ¶/fig/table/other)* |
| --- | --- | --- |
| Group name |  |  |
| No. randomised to group  *(specify whether no. people or clusters)* |  |  |
| Theoretical basis *(include key references)* |  |  |
| Description *(include sufficient detail for replication, e.g. content, dose, components)* |  |  |
| Duration of treatment period |  |  |
| Timing *(e.g. frequency, duration of each episode)* |  |  |
| Delivery *(e.g. study visit, lecture, webpage etc.)* |  |  |
| Providers  *(e.g. a nurse, nutritionist, student, webpage etc.)* |  |  |
| Co-interventions |  |  |
| Economic information *(i.e. intervention cost, changes in other costs as result of intervention)* |  |  |
| Resource requirements  *(e.g. staff numbers, cold chain, equipment)* |  |  |
|  |  |  |
| Compliance |  |  |
| Notes: | | |

## Outcomes

Those outcomes that are relevant to answer our research question!

*Copy and paste table for each outcome.*

**Outcome 1**

|  | Description as stated in report/paper | | Location in text or source *(pg & ¶/fig/table/other)* |
| --- | --- | --- | --- |
| Outcome name |  | |  |
| Time points measured  *(specify whether from start or end of intervention)* |  | |  |
| Time points reported |  | |  |
| Outcome definition *(with diagnostic criteria if relevant)* |  | |  |
| Person measuring/ reporting |  | |  |
| Unit of measurement  *(if relevant)* |  | |  |
| Scales: upper and lower limits *(indicate whether high or low score is good)* |  | |  |
| Is outcome/tool validated? | Yes No Unclear |  |  |
| Imputation of missing data *(e.g. assumptions made for ITT analysis)* | No | |  |
| Assumed risk estimate, i.e. risk estimate without intervention  *(e.g. baseline or population risk noted in Background)* |  | |  |
| Power *(e.g. power & sample size calculation, level of power achieved)* |  | |  |
| Notes: | | | |

## Other

| **Study funding sources**  *(including role of funders)* |  |  |
| --- | --- | --- |
| **Possible conflicts of interest**  *(for study authors)* |  |  |
| **Notes:** | | |

# Risk of Bias assessment

Risk of bias “cribsheet”:

<https://drive.google.com/file/d/1Q4Fk3HCuBRwIDWTGZa5oH11OdR4Gbhdo/view>

Full guidance:

<https://drive.google.com/file/d/19R9savfPdCHC8XLz2iiMvL_71lPJERWK/view>

*See* [*Chapter 8*](http://www.mrc-bsu.cam.ac.uk/cochrane/handbook/index.htm#chapter_8/8_assessing_risk_of_bias_in_included_studies.htm) *of the Cochrane Handbook. Additional domains may be added for non-randomised studies.*

| Domain | Risk of bias | | | Support for judgement  *(include direct quotes where available with explanatory comments)* | Location in text or source *(pg & ¶/fig/table/other)* |
| --- | --- | --- | --- | --- | --- |
|  | Low | High | Unclear |  |  |
| Random sequence generation  *(selection bias)*  *(e.g. computer-generated random numbers; reference to a random number table; coin tossing; shuffling cards or envelopes; throwing dice; or drawing lots)* |  |  |  |  |  |
| Allocation concealment  The person doing the randomization is not aware of the allocation sequence  *(selection bias)*  *Examples of appropriate allocation concealment: allocation controlled by an external unit independent of the enrolment personnel (e.g. independent central pharmacy, telephone or internet-based randomization service providers).*  *OR*  *Appropriately used envelopes: Envelopes should be opaque, sequentially numbered, sealed and opened only after the envelope has been irreversibly assigned to the participant.* |  |  |  |  |  |
| Blinding of participants and personnel  *(performance bias)*  *(In our case, usually not blinded -> high risk of bias)* |  |  |  | Outcome group: All/ |  |
| *(if separate judgement by outcome(s) required)* |  |  |  | Outcome group: |  |
| Blinding of outcome assessment  *(detection bias)* |  |  |  | Outcome group: All/ |  |
| *(if separate judgement by outcome(s) required)* |  |  |  | Outcome group: |  |
| Incomplete outcome data^1^  *(attrition bias)*  *Missingness of outcome data is likely to result from the value of the outcome, e.g. less motivated people are not motivated to finish the study -> tool had probably less impact on their diet* |  |  |  | Outcome group: All/ |  |
| *(if separate judgement by outcome(s) required)* |  |  |  | Outcome group: |  |
| Selective outcome reporting?^2^  *If reported differently than in the protocol description*  *(reporting bias)* |  |  |  |  |  |
| Other bias |  |  |  |  |  |
| Notes: | | | | | |

# Data and analysis

*Copy and paste the appropriate table for each outcome, including additional tables for each time point and subgroup as required.*

***Dichotomous outcome***

|  | Description as stated in report/paper | | | | | Location in text or source *(pg & ¶/fig/table/other)* |
| --- | --- | --- | --- | --- | --- | --- |
| Comparison |  | | | | |  |
| Outcome |  | | | | |  |
| Subgroup |  | | | | |  |
| Time point *(specify from start or end of intervention)* |  | | | | |  |
| Results | Intervention | | | Comparison | |  |
|  | No. with event | Total in group | | No. with event | Total in group |  |
|  |  |  | |  |  |  |
| Any other results reported *(e.g. odds ratio, risk difference, CI or P value)* |  | | | | |  |
| No. missing participants |  | | |  | |  |
| Reasons missing |  | | |  | |  |
| No. participants moved from other group |  | | |  | |  |
| Reasons moved |  | | |  | |  |
| Unit of analysis *(by individuals, cluster/groups or body parts)* |  | | | | |  |
| Statistical methods used and appropriateness of these *(e.g. adjustment for correlation)* |  | | | | |  |
| Reanalysis required? *(specify, e.g. correlation adjustment)* | Yes No Unclear | |  | | |  |
| Reanalysis possible? | Yes No Unclear | |  | | |  |
| Reanalysed results |  | | | | |  |
| Notes: | | | | | | |

***For RCT/CCT***

***Continuous outcome***

|  | | Description as stated in report/paper | | | | | Location in text or source *(pg & ¶/fig/table/other)* | |
| --- | --- | --- | --- | --- | --- | --- | --- | --- |
| Comparison | |  | | | | |  | |
| Outcome | |  | | | | |  | |
| Subgroup | |  | | | | |  | |
| Time point *(specify from start or end of intervention)* | |  | | | | |  | |
| Post-intervention or change from baseline? | |  | | | | |  | |
| Results | Intervention | | | Comparison | | |  | |
|  | Mean | SD *(or other variance, specify)* | No. participants | Mean | SD *(or other variance, specify)* | No. participants |  |  |
|  |  |  |  |  |  |  |  |  |
| Any other results reported *(e.g. mean difference, CI, P value)* | |  | | | | |  | |
| No. missing participants | |  | |  | | |  |  |
| Reasons missing | |  | |  | | |  |  |
| No. participants moved from other group | |  | |  | | |  |  |
| Reasons moved | |  | |  | | |  |  |
| Unit of analysis  *(individuals, cluster/ groups or body parts)* | |  | | | | |  | |
| Statistical methods used and appropriateness of these *(e.g. adjustment for correlation) OR CONFOUNDING* | |  | | | | |  | |
| Reanalysis required? *(specify)* | | Yes No Unclear | |  | | |  | |
| Reanalysis possible? | | Yes No Unclear | |  | | |  | |
| Reanalysed results | |  | | | | |  | |
| Notes: | | | | | | | | |

***Continuous outcome***

|  | | Description as stated in report/paper | | | | | | Location in text or source *(pg & ¶/fig/table/other)* | |
| --- | --- | --- | --- | --- | --- | --- | --- | --- | --- |
| Comparison | |  | | | | | |  | |
| Outcome | |  | | | | | |  | |
| Subgroup | |  | | | | | |  | |
| Time point *(specify from start or end of intervention)* | |  | | | | | |  | |
| Post-intervention or change from baseline? | |  | | | | | |  | |
| Results | Intervention | | | | Comparison | | |  | |
|  | Mean | SD *(or other variance, specify)* | No. participants | | Mean | SD *(or other variance, specify)* | No. participants |  | |
|  |  |  |  | |  |  |  |  |  |
| Any other results reported *(e.g. mean difference, CI, P value)* | |  | | | | | |  | |
| No. missing participants | |  | | |  | | |  |  |
| Reasons missing | |  | | |  | | |  |  |
| No. participants moved from other group | |  | | |  | | |  |  |
| Reasons moved | |  | | |  | | |  |  |
| Unit of analysis  *(individuals, cluster/ groups or body parts)* | |  | | | | | |  | |
| Statistical methods used and appropriateness of these *(e.g. adjustment for correlation)* | |  | | | | | |  | |
| Reanalysis required? *(specify)* | | Yes No Unclear | |  | | | |  | |
| Reanalysis possible? | | Yes No Unclear | |  | | | |  | |
| Reanalysed results | |  | | | | | |  | |
| Notes: | | | | | | | | |  |

***Other outcome***

|  | Description as stated in report/paper | | | | | Location in text or source *(pg & ¶/fig/table/other)* |
| --- | --- | --- | --- | --- | --- | --- |
| Comparison |  | | | | |  |
| Outcome |  | | | | |  |
| Subgroup |  | | | | |  |
| Time point *(specify from start or end of intervention)* |  | | | | |  |
| No. participants | Intervention | | | Control | |  |
|  |  | | |  | |  |
| Results | Intervention result | SE (or other variance) | | Control result | SE (or other variance) |  |
|  |  |  | |  |  |  |
|  | Overall results | | | SE (or other variance) | |  |
|  |  | | |  | |  |
| Any other results reported |  | | | | |  |
| No. missing participants |  | | |  | |  |
| Reasons missing |  | | |  | |  |
| No. participants moved from other group |  | | |  | |  |
| Reasons moved |  | | |  | |  |
| Unit of analysis *(by individuals, cluster/groups or body parts)* |  | | | | |  |
| Statistical methods used and appropriateness of these |  | | | | |  |
| Reanalysis required? *(specify)* | Yes No Unclear | |  | | |  |
| Reanalysis possible? | Yes No Unclear | |  | | |  |
| Reanalysed results |  | | | | |  |
| Notes: | | | | | | |

# Other information

|  | **Description as stated in report/paper** | **Location in text or source** *(pg & ¶/fig/table/other)* |
| --- | --- | --- |
| **Key conclusions of study authors** |  |  |
| **References to other relevant studies** |  |  |
| **Correspondence required for further study information** *(from whom, what and when)* |  | |
| **Notes:** | | |

# Definitions

| Assumed risk estimate | An estimate of the risk of an event or average score without the intervention, used in Cochrane 'Summary of findings tables'. If a study provides useful estimates of the risk or average score of different subgroups of the population, or an estimate based on a representative observational study, you may wish to collect this information. |
| --- | --- |
| Bias | A systematic error or deviation in results or inferences from the truth. In studies of the effects of health care, the main types of bias arise from systematic differences in the groups that are compared (selection bias), the care that is provided, exposure to other factors apart from the intervention of interest (performance bias), withdrawals or exclusions of people entered into a study (attrition bias) or how outcomes are assessed (detection bias). Reviews of studies may also be particularly affected by reporting bias, where a biased subset of all the relevant data is available. |
| Change from baseline | A measure for a continuous outcome calculated as the difference between the baseline score and the post-intervention score. |
| Clusters | A group of participants who have been allocated to the same intervention arm together, as in a cluster-randomised trial, e.g. a whole family, town, school or patients in a clinic may be allocated to the same intervention rather than separately allocating each individual to different arms. |
| Co-morbidities | The presence of one or more diseases or conditions other than those of primary interest. In a study looking at treatment for one disease or condition, some of the individuals may have other diseases or conditions that could affect their outcomes. |
| Compliance | Participant behaviour that abides by the recommendations of a doctor, other health care provider or study investigator (also called adherence or concordance). |
| Contemporaneous data collection | When data are collected at the same point(s) in time or covering the same time period for each intervention arm in a study (that is, historical data are not used as a comparison). |
| Exclusions | Participants who were excluded from the study or the analysis by the investigators. |
| Imputation | Assuming a value for a measure where the true value is not available (e.g. assuming last observation carried forward for missing participants). |
| Integrity of delivery | The degree to which the specified procedures or components of an intervention are delivered as originally planned. |
| Post-intervention | The value of an outcome measured at some time point following the beginning of the intervention (may be during or after the intervention period). |
| Power | In clinical trials, power is the probability that a trial will obtain a statistically significant result when the true intervention effect is a specified size. For a given size of effect, studies with more participants have greater power. Note that power should not be considered in the risk of bias assessment. |
| Providers | The person or people responsible for delivering an intervention and related care, who may or may not require specific qualifications (e.g. doctors, physiotherapists) or training. |
| Quasi-randomised controlled trial | A study in which the method of allocating people to intervention arms was not random, but was intended to produce similar groups when used to allocate participants. Quasi-random methods include: allocation by the person's date of birth, by the day of the week or month of the year, by a person's medical record number, or just allocating every alternate person. |
| Reanalysis | Additional analysis of a study's results by a review author (e.g. to introduce adjustment for correlation that was not done by the study authors). |
| Report ID | A unique ID code given to a publication or other report of a study by the review author (e.g. first author's name and year of publication). If a study has more than one report (e.g. multiple publications or additional unpublished data) a separate Report ID can be allocated to each to help review authors keep track of the source of extracted data. |
| Sociodemographics | Social and demographic information about a study or its participants, including economic and cultural information, location, age, gender, ethnicity, etc. |
| Study ID | A unique ID code given to an included or excluded study by the review author (e.g. first author's name and year of publication from the main report of the study). Although a study may have multiple reports or references, it should have one single Study ID to help review authors keep track of all the different sources of information for a study. |
| Theoretical basis | The use of a particular theory (such as theories of human behaviour change) to design the components and implementation of an intervention |
| Unit of allocation | The unit allocated to an intervention arm. In most studies individual participants will be allocated, but in others it may be individual body parts (e.g. different teeth or joints may be allocated separately) or clusters of multiple people. |
| Unit of analysis | The unit used to calculate N in an analysis, and for which the result is reported. This may be the number of individual people, or the number of body parts or clusters of people in the study. |
| Unit of measurement | The unit in which an outcome is measured, e.g. height may be measured in cm or inches; depression may be measured using points on a particular scale. |
| Validation | A process to test and establish that a particular measurement tool or scale is a good measure of that outcome. |
| Withdrawals | Participants who voluntarily withdrew from participation in a study before the completion of outcome measurement. |

^1^ How to spot incomplete outcome data:

1. Differences between intervention groups in the proportions of missing outcome data. If there is a difference between the effects of the experimental and comparator interventions on the outcome, and the missingness in the outcome is influenced by its true value, then the proportions of missing outcome data are likely to differ between intervention groups. Such a difference suggests a risk of bias due to missing outcome data, because the trial result will be sensitive to missingness in the outcome being related to its true value. For time-to-event-data, the analogue is that rates of censoring (loss to follow-up) differ between the intervention groups.

2. Reported reasons for missing outcome data provide evidence that missingness in the outcome depends on its true value;

3. Reported reasons for missing outcome data differ between the intervention groups;

4. The circumstances of the trial make it likely that missingness in the outcome depends on its true value. For example, in trials of interventions to treat schizophrenia it is widely understood that continuing symptoms make drop out more likely.

5. In time-to-event analyses, participants’ follow up is censored when they stop or change their assigned intervention, for example because of drug toxicity or, in cancer trials, when participants switch to second-line chemotherapy.

^2^Selective outcome reporting examples:

Example: if post-intervention diet quality has been measured at 6, 12, and 24 months from baseline and only result from 6 months is reported

Example 2: if in the protocol/plan of the study different outcomes are listed than the outcomes eventually reported.

Sources:

Cochrane Collaboration Glossary, 2010. Available from <http://www.cochrane.org/training/cochrane-handbook>.

Higgins JPT, Green S (editors). Cochrane Handbook for Systematic Reviews of Interventions Version 5.1.0 [updated March 2011]. The Cochrane Collaboration, 2011. Available from [www.cochrane-handbook.org](http://www.cochrane-handbook.org).

Last JM (editor), A Dictionary of Epidemiology, 4^th^ Ed. New York: Oxford University Press, 2001.

Schünemann H, Brożek J, Oxman A, editors. GRADE handbook for grading quality of evidence and strength of recommendation. Version 3.2 [updated March 2009]. The GRADE Working Group, 2009. Available from <http://www.cc-ims.net/gradepro>.
